# Dyslipidemia and Cardiovascular Disease: Current Knowledge, Existing Challenges, and New Opportunities for Management Strategies

**DOI:** 10.3390/jcm12010363

**Published:** 2023-01-03

**Authors:** Zhiyong Du, Yanwen Qin

**Affiliations:** 1 The Key Laboratory of Remodeling-Related Cardiovascular Diseases, Ministry of Education, National Clinical Research Center for Cardiovascular Diseases, Beijing Anzhen Hospital, Capital Medical University, Beijing 100029, China; 2 Beijing Institute of Heart Lung and Blood Vessel Disease, Beijing 100029, China

Cardiovascular disease is the leading cause of morbidity and mortality worldwide, and dyslipidemia is one of the major risk factors. Hypercholesterolemia is the most common form of dyslipidemia [1]. Low-density lipoprotein (LDL), as the most abundant apolipoprotein B (ApoB)-containing lipoprotein in human plasma, is the key transmitter of cholesterol to the vascular artery wall. The elevation of LDL cholesterol is the principal aspect of dyslipidemia and associated with an increased cardiovascular risk, particularly in atherosclerotic cardiovascular disease (ASCVD) [1,2]. Numerous epidemiologic, clinical, and experimental studies have posited the pivotal roles of LDL cholesterol and its oxidized form as the primary driving forces of atherosclerosis progression [3,4]. Therefore, lowering the LDL cholesterol levels is one of the most prevalent strategies for the treatment and prevention of ASCVD in clinical practice [5].

Highly efficacious lipid-lowering therapies can lower LDL cholesterol and have been associated with a decrease in cardiovascular morbidity and mortality in randomized controlled trials [6,7]. Currently, statins are the first choice for LDL cholesterol lowering in most clinical settings. Ezetimibe and hypertime, the Niemann-Pick C1-Like 1 (NPC1L1) inhibitors, have generally been used as complement therapies to statins when patients cannot meet their treatment goals [8]. However, a substantial proportion of patients cannot achieve an ideal LDL cholesterol concentration with the use of statin treatments, even in combination with NPC1L1 inhibitors [7,9]. Furthermore, statins can cause muscle-related side effects in a certain proportion of patients [10]. In the past decade, several non-statin lipid-lowering agents have emerged.

Proprotein convertase subtilisin/kexin type 9 (PCSK9), a key protein mediating hepatic LDL receptor degradation, has emerged as a novel target for the lowering of the LDL cholesterol levels. Two clinically available anti-PCSK9 monoclonal antibodies, evolocumab and alirocumab, can offer greater reductions (50–60%) than those feasible through the application of NPC1L1 inhibitors [11]. However, their high cost and delivery by injection hamper their widespread use [12]. Currently, non-antibody approaches to the inhibition of PCSK9 function or gene expression (such as antisense oligonucleotides, genome editing, and vaccination) are in the advanced phases of development. If proven to lower LDL cholesterol and decrease cardiovascular events in clinical trials, these new therapeutic agents may confer promising advantages over anti-PCSK9 antibodies, including an improved durability, more convenient dosage regimens, and, possibly, cost-effectiveness [7,12].

Bempedoic acid is another new agent that functions by blocking adenosine triphosphate-citrate lyase, a cytosolic enzyme upstream of the hydroxymethylglutaryl coenzyme (target of statins) in the cholesterol biosynthesis pathway [13]. In several clinical trials, its use as a monotherapy or in combination with conventional lipid-lowering therapies led to significantly lower LDL cholesterol [14,15]. More recently, the United States Food and Drug Administration and European Medicines Agency approved bempedoic acid for the treatment of hypercholesterolemia [13,14,15].

In addition to LDL cholesterol, a wealth of evidence suggests that other ApoB-containing lipoproteins can also causally contribute to the development of cardiovascular diseases, including lipoprotein(a) (Lp(a))- and triglyceride-rich lipoproteins [16,17,18]. Lp(a) is an LDL–like particle characterized by the covalent addition of a highly polymorphic apolipoprotein(a) to ApoB via a thioester bond [19]. Previous clinical and genetic studies have shown that elevated Lp(a) levels can increase the ASCVD risk, independent of LDL cholesterol [19,20]. However, the current treatment options for elevated Lp(a) are limited. Conventional lipid-lowering drugs have little or no effect on the Lp(a) levels [21]. The novel PCSK9 inhibitor, evolocumab, can only reduce Lp(a) by approximately 20% to 30% [22]. Olpasiran is an acetylgalactosamine-conjugated small interfering RNA designed to interrupt hepatic lipoprotein(a) synthesis [23]. More recently, a clinical phase 2 study demonstrated that olpasiran can substantially reduce the Lp(a) levels by more than 90% [24]. It will be interesting to observe whether this new agent for lowering Lp(a) concentrations can improve cardiovascular outcomes in the future studies.

Elevated levels of triglyceride and triglyceride-rich lipoproteins are also important cardiovascular risk factors [17,18,25,26]. Several efficacious and cost-effective triglyceride-lowering agents are widely used in the clinic, including fibrates, niacin, and omega-3 fatty acids [26]. The measurement of the plasma triglyceride levels serves a biomarker for a class of triglyceride-rich lipoproteins, including chylomicron remnants, very low-density lipoprotein (VLDL), and intermediate-density lipoprotein (IDL). Triglyceride-rich lipoproteins can traverse the endothelium, accumulate, and promote atherosclerosis progression. Lipoprotein lipase (LPL) is the primary plasma enzyme mediating triglyceride degradation. The lower activity or levels of LPL can cause increased concentrations of triglyceride and triglyceride-rich lipoproteins [27]. Genetic and functional studies have identified several other proteins linked to LPL that have a regulatory role in determining the levels of triglyceride-rich lipoproteins, mainly apolipoprotein C (APOC) and angiopoietin-like protein (ANGPTL). Volanesorsen is an antisense oligonucleotide involved in apolipoprotein C3 synthesis in hepatocytes. In a phase 3 trial, it was found to reduce the plasma triglyceride levels by approximately 70% [28]. Evinacumab (a monoclonal antibody) and vupanorsen (an antisense oligonucleotide) are newly designed agents that function by inhibiting ANGPTL3. These inhibitors have shown great effectiveness in the reduction of circulating triglycerides, LDL cholesterol, and triglyceride-rich lipoproteins in several clinical trials [29,30,31].

Unlike cholesterol-rich LDL or triglyceride-rich lipoproteins, apolipoprotein AI-enriched high-density lipoprotein (HDL) cholesterol is well documented to correlate negatively with the ASCVD risk [32]. The possible antiatherogenic effects ascribed to HDL cholesterol include the promotion of anti-inflammatory and anti-thrombotic actions, prevention of oxidative stress, and mediation of cholesterol efflux from the macrophages [32,33]. In recent decades, raising the HDL cholesterol levels has been considered as a promising therapeutic strategy for the prevention and management of ASCVD. However, accumulating clinical data demonstrate that increasing the HDL cholesterol levels fails to show a positive impact on cardiovascular outcomes and may increase the non-cardiovascular disease risk [33,34]. Recent evidence suggests that future therapies targeting HDL should focus on improving its quality, in addition to raising its levels [35].

Collectively, our increased understanding of dyslipidemia and advances in pharmaceutical technologies have provided a diversity of novel therapeutic targets and lipid-lowering agents for the prevention of cardiovascular diseases. Several newly approved agents with novel mechanisms of action have shown promising potential to significantly reduce LDL cholesterol concentrations. However, most of the new lipid-lowering therapies that target other causal lipoproteins are still undergoing experimental or clinical testing. Further detailed clinical confirmation and evaluations will help us to determine the safest, most efficacious, and most cost-effective agents that may be used to improve cardiovascular outcomes and decrease the growing burden of ASCVD.

We invite researchers to submit novel research articles, state-of-the-art reviews, and communications related to the fields of dyslipidemia and cardiovascular disease to this Special Issue. Such papers can focus on novel lipid-lowering strategies, cardiovascular outcomes after intervention, the primary and secondary prevention of cardiovascular diseases, precise diagnosis of different forms of dyslipidemia, ASCVD risk stratification, and more. We also welcome pre-clinical papers focusing on the atherosclerotic actions of small-molecule metabolite or lipid alterations, such as ceramides, lysophospholipids, and gut microbiota derivatives. We look forward to receiving your contributions and to future collaborations.
